# Clinical Characteristics of Patients With Respiratory Infections After Nonpharmacological Interventions for COVID‐19 in China Have Ended: Using Machine Learning Approaches to Support Pathogen Prediction at Admission

**DOI:** 10.1002/iid3.70237

**Published:** 2025-08-08

**Authors:** Tian‐ning Li, Yan‐hong Liu, Kwok‐Leung Yiu, Lu Liu, Meng Han, Wei‐jia Ma, Chun‐lei Zhou, Hong Mu

**Affiliations:** ^1^ Department of Clinical Lab Tianjin First Central Hospital Tianjin China; ^2^ Tianjin Union Medical Center Nankai University Tianjin China; ^3^ Roche Diagnostics Shanghai China

**Keywords:** influenza A, machine learning predictive model, *Mycoplasma pneumoniae*, respiratory pathogens

## Abstract

**Objectives:**

In the aftermath of the COVID‐19 pandemic, China witnessed a surge in respiratory virus infections, which presented considerable challenges to primary health care systems. This study developed an interpretable prediction model using complete blood count (CBC) test data. This model aims to identify common respiratory virus infections in patients.

**Methods:**

The study's derivation cohort included 7471 patients who presented with fever at Central Hospital between November and December 2023. Each patient underwent diagnostic procedures, including influenza A (Flu A) and *Mycoplasma pneumoniae* (MP) antibody testing and CBC. On the basis of the results of the CBC and patients' basic information, modelling and prediction through machine learning (ML) were performed, and external verification was conducted.

**Results:**

Among the developed models, we constructed two distinct versions of the three‐class model: one emphasizing high recall and the other balancing precision and recall. The final model was refined through manual parameter adjustments and a comprehensive network search. The high‐recall model demonstrated superior performance in detecting Flu A, with a recall rate of 81.0%. Conversely, the precision‒recall balanced model exhibited enhanced accuracy in identifying MP cases, with a precision rate of 84.3%.

**Conclusion:**

Our interpretable ML model not only achieves accurate identification of Flu A and MP infections in febrile patients but also addresses the prevalent “black box” concerns associated with ML techniques. This technique can aid clinicians in enhancing diagnostic efficiency and accuracy. Therefore, this improvement can lead to reduced medical expenses by minimizing unnecessary tests and treatments.

## Graphical abstract

We have analyzed the medical records of 7471 patients presenting with fever at Tianjin First Central Hospital. Our study successfully developed a predictive model which was able to make its predictions. This robust model effectively identifies influenza A (flu A) and MP infections in febrile individuals. Its accuracy is pivotal in enabling swift and accurate diagnostic classification in primary healthcare settings.

## 1 Introduction

The extensive influenza outbreak post‐COVID‐19 has presented formidable challenges to primary health systems, especially in developing countries [1]. Additionally, the substantial cost associated with viral testing has become an economic strain for many individuals. To understand the current landscape of research in this field, we conducted a comprehensive literature review on PubMed, employing “respiratory pathogens” and “model prediction” as our primary search terms, without any language constraints. Notably, the majority of the literature concentrates on forecasting influenza trends [2, 3]. Our research stands in that it establishes an interpretable predictive model [4, 5] specifically designed for the identification of respiratory pathogen infections in patients with fever. This gap in existing research underscores the novelty and potential impact of our study in the realm of infectious disease diagnostics [6, 7]. We hope to developet models that reduce medical costs, the pressure on frontline medical staff, the use of “defensive” medical methods and the overprescription of antibiotics.

## 2 Methods

### 2.1 Study Design and Data Acquisition

**Figure 1:**
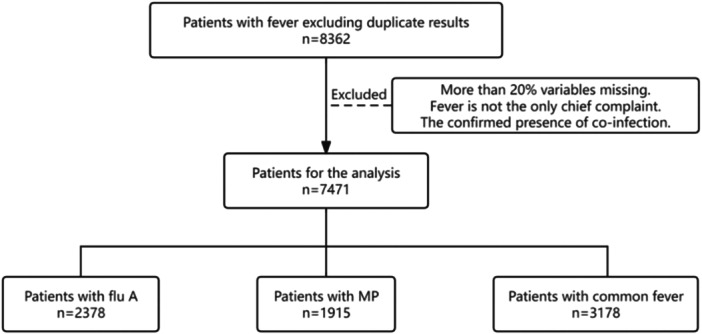
Flowchart depicting steps in obtaining the data set.

A retrospective study was carried out in the Emergency Department of Tianjin First Central Hospital from November 1, 2023, to December 1, 2023. The diagnosis of Flu A and MP was performed via a colloidal gold assay (Lizhu Company, Zhuhai, Guangdong, China). As the study involved only anonymized patient data, with no analysis of personally identifiable information, the requirement for additional informed consent was waived. A flowchart detailing patient enrolment is included in Figure 1.

### 2.2 Data Preprocessing

The study analysed a deidentified data set comprising 7471 patients. A total of twenty‐seven variables were collected, including one continuous variable, age, and four categorical variables: sex, Flu A, MP, and common fever. The age variable was uniformly converted to a decimal format, with “years” as the measurement unit.

1. Data cleaning: Handling missing values: Samples with missing gender or age data were removed (0.15% of original data). Gender encoding: Male mapped to 1, female to 0. Age standardization: All ages converted to decimal years. Sample filtering: Newborns < 28 days old and cases with multiple infections were excluded.
2. Feature engineering: Feature binning: We employed three strategies ‐ reference interval binning, outlier subdivision, and equal‐frequency binning [8]. Feature derivation: Statistical values were derived for each indicator. Feature combination: We used variance analysis to identify effective feature combinations. Feature selection: Correlation filtering and ensemble tree model feature importance were used to select the final 44 features.

We categorized 17 haematological parameters, such as white blood cells (WBCs) and red blood cells (RBCs), into three groups: “low,” “medium,” and “high.” For indicators not included in these reference intervals, such as RDW‐SD, MPV, P‐LCR, PCT, IG%, and IG#, corresponding intervals were sourced from medical literature and classified using the same rules.

In addition to these categorizations, age was divided into 10 specific groups to align with the age divisions indicated in the reference intervals. For those indicators that surpassed the upper limit of the established reference interval, an additional subcategorization was implemented, dividing them into “high” and “very high” groups. Furthermore, for eight specific indicators—LYBL%, NRBC%, RDW‐CV, PDW, HFLC1, CRP, HFLC2, and NRBC#—which were not addressed by the previous binning strategies, equal‐frequency binning was also employed. In addition to these categorization methods, this study further enhances the analytical robustness of each indicator by deriving statistical values, thereby introducing new features.

### 2.3 Data Characterization Screening

We took steps to identify and eliminate potential redundancy among the features. Any feature exhibiting a correlation coefficient greater than 0.95 was excluded from further analysis.

To develop robust predictive algorithms, we trained three different machine learning models: LightGBM [9], CatBoost [10], and XGBoost [11]. The significance of each feature was assessed through feature importance scores generated by each model. These scores were then aggregated, assigning different weights on the basis of the F1 performance of each model when applied to our data set. The weighting scheme was as follows: LightGBM was assigned a weight of 0.7, CatBoost was given a weight of 0.1, and XGBoost was allocated a weight of 0.2.

Each set was independently input into an ensemble gradient boosting tree model. From each set, the 14 most influential features were identified on the basis of their importance scores. All the selected features from these sets were subsequently pooled and reintroduced into the ensemble model for a final analysis. This approach culminated in a refined feature set comprising 56 unique features after accounting for and eliminating any duplicates.

### 2.4 Algorithm Training and Validation

To maintain a consistent distribution of data between the training and testing datasets, a stratified split was utilized, allocating 80% of the data for training and 20% for testing.

This study involved a comprehensive comparative analysis of the three leading gradient boosting tree models. To effectively address the issue of class imbalance in our data set, a strategy of weighting the minority class samples was adopted. The weights assigned to each class were fine‐tuned on the basis of insights obtained from exploratory data analysis (EDA) and iterative feedback during the model development process. Consequently, the final class weights were established as follows: 1.0 for common fever, 1.18 for MP, and 1.4 for Flu A.

Additionally, to counteract the potential for overfitting, several measures were implemented during the training of the LightGBM model. The feature sampling rate was set at 0.5. Regularization penalties were introduced as an additional safeguard.

### 2.5 Evolution of the Stability of the Model

To measure the stability of the model, the population stability index (PSI) indicator is employed. The calculation formula for the PSI is as follows:

$$PSI=\sum_{i=1}^{n}\left(P_{train,t}-P_{test,i}\right)\times \text{In}\left(\frac{P_{train,t}}{P_{test,i}}\right).$$

### 2.6 Statistical Analysis

Group variables are represented by numbers (%), normally distributed continuous variables are represented by means ± standard deviations (SDs), and nonnormally distributed continuous variables are represented by medians (interquartile ranges). Nonnormally distributed continuous variables included laboratory indicators. SPSS 22.0 software (version 22.0; IBM Corp., Armonk, NY, USA) was used for all the statistical analyses. All tests were two sided, and *p* values of < 0.05 were considered statistically significant.

## 3 Results

### 3.1 Baseline Characteristics of the Study Populations

A total of 7471 patients were included, including 1915 patients infected with MP, 2378 patients infected with Flu A, and 3178 patients with general fever. The detailed baseline characteristics and laboratory indicators of these patients are systematically outlined in Table 1. Notably, a greater proportion of female patients were diagnosed with MP than with Flu A or general fever (60.99% vs. 53.36% vs. 54.94%, *p* < 0.01). A distinct age trend was observed in the patient populations. The average age of patients with MP infection was 12.68 years, which was substantially younger than the 26.72‐year average age of Flu A patients. Children are the main susceptible population for MP infection.

**Table 1:** Baseline characteristics of enrolled patients.

	Flu A (*n* = 2378)	MP (*n* = 1915)	Common fever (*n* = 3178)	*p* value
**Sex**				< 0.001*
Male	1109 (46.64%)	747 (39.01%)	1432 (45.06%)	
Female	1269 (53.36%)	1168 (60.99%)	1746 (54.94%)	
**Age range (years)**				< 0.001*
0–17 (juvenile)	311 (13.08%)	1322 (69.03%)	461 (14.51%)	
18–44 (youth)	1707 (71.78%)	547 (28.56%)	2227 (70.08%)	
45–59 (middle‐aged)	131 (5.51%)	24 (1.25%)	202 (6.36%)	
≥ 60 (elderly)	229 (9.63%)	22 (1.15%)	288 (9.06%)	

### 3.2 Differences in Laboratory Indicators Between Patients With Flu A, MP and Common Fever

We conducted a detailed comparative analysis of routine blood cell analysis results across three patient categories. The findings of this analysis are comprehensively presented in Table 2. The results revealed significant differences among these three groups in terms of platelet, neutrophil, lymphocyte, and monocyte counts. Specifically, Flu A patients presented lower levels of platelets, neutrophils, and lymphocytes than did the other two groups. However, the monocyte count in Flu A patients was significantly greater than that in the other two groups (0.74 vs. 0.65 vs. 0.69).

**Table 2:** Differences of laboratory indicators between patients with flu A, MP and Common Fever.

	Flu A (*n* = 2378)	MP (*n* = 1915)	Common fever (*n* = 3178)
White‐cell count (×10^9^/L)	7.02 (5.45, 8.17)	8.47 (5.9, 10.13)	7.92 (5.56, 9.42)
Red‐cell count (×10^12^/L)	4.66 (4.34, 5.04)	4.66 (4.36, 4.93)	4.68 (4.32, 5.02)
Hemoglobin (g/L)	139.1 (129, 152)	134.07 (125, 142)	139.88 (130, 152)
Hematocrit (%)	41.58 (38.9, 45.2)	40.34 (37.8, 42.7)	41.63 (38.7, 44.9)
Mean corpuscular volume (fL)	89.39 (87.2, 92.3)	86.73 (83.9, 90)	89.21 (87.1, 92.2)
Mean corpuscular hemoglobin (pg)	29.9 (29.1, 31.2)	28.82 (27.8, 30.2)	29.98 (29.2, 31.2)
Mean corpuscular hemoglobin concentration (g/L)	334.23 (329, 341)	332.14 (327, 338)	335.8 (331, 342)
Platelet (×10^9^/L)	215.57 (179, 248)	265.5 (210, 310)	228.33 (186, 261)
Standard deviation in red cell distribution width (fL)	42.33 (40.1, 43.8)	41.54 (39.8, 42.9)	42.1 (40.3, 43.6)
Coefficient variation of red blood cell volume distribution width (%)	12.92 (12.1, 13.2)	13.15 (12.5, 13.5)	12.96 (12.3, 13.3)
Platelet distribution width (fL)	13.37 (10.3, 16.1)	15.35 (15.6, 16.2)	14.32 (11.7, 16.3)
Mean platelet volume (fL)	9.61 (9, 10.2)	9.11 (8.4, 9.8)	9.45 (8.8, 10.1)
Platelet larger cell ratio (%)	22.62 (17.6, 26.5)	20.12 (15.2, 24.5)	21.99 (17.2, 26)
Thrombocytocrit (%)	0.21 (0.171, 0.233)	0.24 (0.194, 0.2735)	0.21 (0.18, 0.242)
Neutrophil count (×10^9^/L)	5.07 (3.64, 6.12)	5.56 (3.43, 7.07)	5.84 (3.64, 7.25)
Lymphocyte count (×10^9^/L)	1.13 (0.74, 1.395)	2.12 (1.23, 2.745)	1.31 (0.81, 1.65)
Monocyte count (×10^9^/L)	0.74 (0.52, 0.88)	0.65 (0.45, 0.79)	0.69 (0.49, 0.84)
Eosinophil count (×10^9^/L)	0.05 (0.01, 0.06)	0.12 (0.02, 0.17)	0.06 (0.01, 0.08)
Basophil count (×10^9^/L)	0.02 (0.01, 0.02)	0.01 (0, 0.02)	0.02 (0.01, 0.02)
Neutrophil ratio (%)	71.04 (64.7, 78.8)	63.36 (53.95, 74.3)	71.1 (63.6, 80.3)
Lymphocyte ratio (%)	17.15 (10.9, 21.65)	26.79 (15.95, 35)	18.38 (10.6, 24.1)
Monocyte ratio (%)	10.81 (8, 13)	8.21 (5.9, 9.8)	9.41 (6.7, 11.5)
Eosinophil ratio (%)	0.7 (0.1, 0.8)	1.48 (0.3, 2.1)	0.86 (0.2, 1.1)
Basophil ratio (%)	0.27 (0.1, 0.4)	0.16 (0, 0.2)	0.25 (0.1, 0.3)
Immature granulocyte count (×10^9^/L)	0.02 (0.01, 0.02)	0.02 (0.01, 0.02)	0.02 (0.01, 0.03)
Immature granulocyte ratio (%)	0.27 (0.1, 0.3)	0.2 (0.1, 0.2)	0.26 (0.1, 0.3)
High fluorescence intensity cell count (%)	0.01 (0, 0.01)	0.04 (0.01, 0.05)	0.01 (0, 0.02)
High fluorescence intensity cell ratio (%)	0.19 (0, 0.2)	0.58 (0.1, 0.7)	0.21 (0.1, 0.2)
C‐reactive protein (mg/L)	19.39 (7.04, 24.79)	11.99 (1.63, 13.91)	21.27 (5.4, 26.53)

### 3.3 Model Evaluation

For the predictive modelling, we opted for LightGBM due to its efficacy and constructed two distinct versions of the three‐class model: one emphasizing high recall and the other balancing precision and recall. The average performance metrics of these models, including the recall, precision, and F1 score [12], are detailed in Table 3. Notably, precision and recall often have an inverse relationship; as precision increases, recall typically decreases, and vice versa [13]. In our study, the high‐recall model demonstrated superior performance in detecting Flu A, with a recall rate of 81.0%. This indicates a heightened ability of this model version to correctly identify Flu A cases among the positive samples. Conversely, the precision‒recall balanced model exhibited enhanced accuracy in identifying MP cases, with a precision rate of 84.3%. This model therefore proved more effective in correctly classifying MP cases among the predicted positives.

**Table 3:** The evaluation of predictive model.

Model	Group	Recall	Precision	F1	Case counts
High recall	Flu A	81.0%	57.7%	67.4%	484
	MP	77.5%	70.8%	74.0%	395
	Common fever	44.7%	71.8%	55.1%	615
	Total	67.7%	66.8%	65.5%	1494
Precision‐recall balanced	Flu A	72.5%	61.3%	66.4%	484
	MP	70.9%	84.3%	77.0%	395
	Common fever	64.4%	67.2%	65.8%	615
	Total	69.3%	70.9%	69.7%	1494

### 3.4 Confusion Matrix

**Figure 2:**
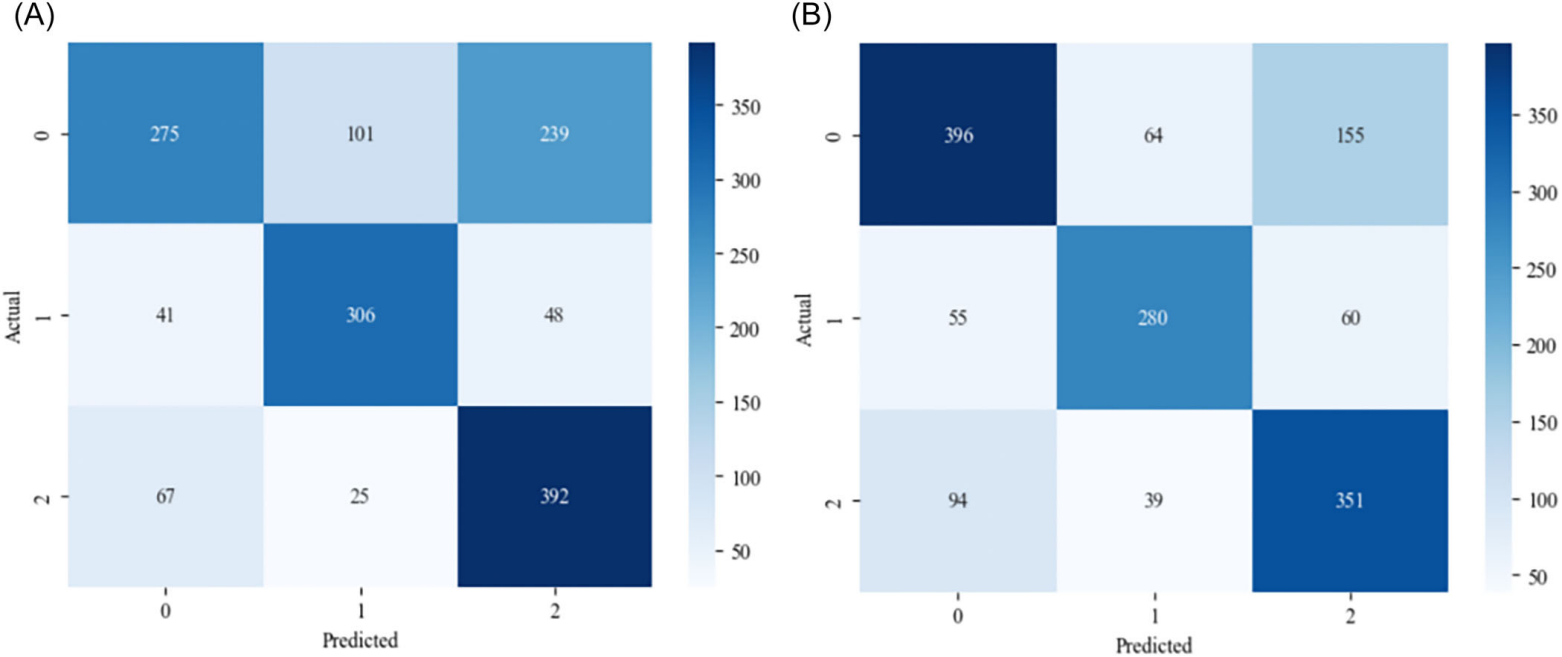
Confusion matrix. (A) High recall model. (B) Precision‐recall balanced model.

The confusion matrices for both the high‐recall version and the precision‒recall balanced version of the model are depicted in Figure 2. For the high‐recall version of the model, which was validated using a test set comprising 1494 cases, the accuracy in identifying different infections was as follows: 392 cases of Flu A were correctly identified, representing an accuracy of 80.99%. For MP infection, 306 cases were accurately diagnosed, accounting for 77.47% of the total cases. For common fever, 275 cases were correctly classified, which constituted a correct identification rate of 44.72%.

On the other hand, the precision‒recall balanced model, which was also validated with the same test set size, showed a different pattern in its classification accuracy. This model correctly identified 351 Flu A cases, resulting in a 70.52% accuracy rate. For MP infection, it accurately diagnosed 280 cases, which translates to 70.89% accuracy. Notably, it was more accurate in identifying common fever, with 396 cases correctly classified, corresponding to a 64.39% accuracy rate.

While the high‐recall model excels in accurately identifying Flu A and MP cases, the balanced model has a stronger ability to correctly diagnose cases of common fever. This variance underscores the importance of selecting an appropriate model on the basis of the specific clinical requirements and the prioritization of either recall or precision in different diagnostic scenarios.

### 3.5 Ealuating and Ranking Feature Importance

**Figure 3:**
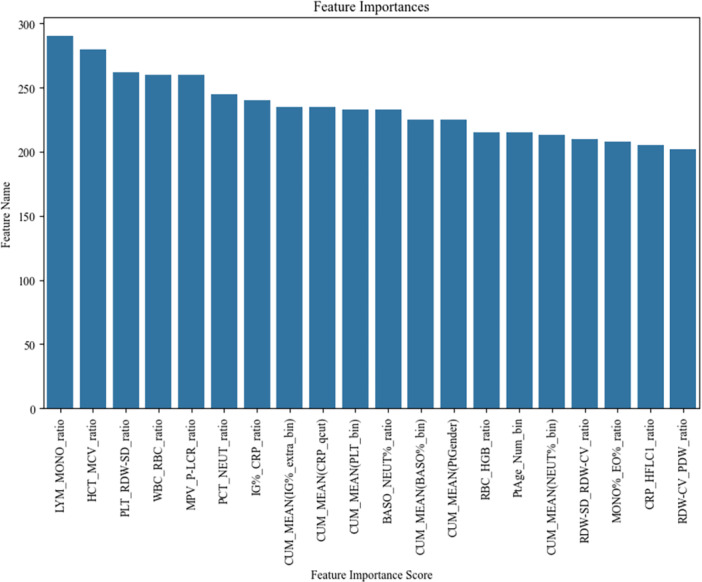
Ealuate and rank feature importance.

Upon detailed analysis, we identified the top five haematological features that significantly contributed to the model's predictive accuracy. These factors were ranked in order of their importance as follows: the lymphocyte‐to‐monocyte ratio (Ly/Mono), the haematocrit‐to‐mean corpuscular volume ratio (HCT/MCV), the platelet count‐to‐red cell distribution width‐standard deviation ratio (PLT/RDW‐SD), the white blood cell‐to‐red blood cell ratio (WBC/RBC), and the mean platelet‐to‐platelet–large cell ratio (MPV/P–LCR). These insights are graphically depicted in Figure 3, where the significance of each feature is evaluated and ranked in a hierarchical order.

### 3.6 Stability of the Model

The stability of the predictive model was rigorously evaluated using the population stability index (PSI). In this analysis, we set “n” to 10 for calculating PSI values across the three patient groups: Flu A, MP, and common fever. The PSI values obtained were 0.0354 for the Flu A group, 0.0122 for the MP group, and 0.0475 for the common fever group. Notably, a lower PSI value is indicative of better model stability, reflecting consistent performance across different data samples.

### 3.7 Analysis of the Missed Data

To understand the limitations of our model, particularly in the context of missed detections, we analysed the model's recall rate for positive samples. The recall rate of 87.7% indicates that 12.3% of the positive cases of Flu A or MP were not successfully identified by the model. This subset of false‐negatives was subjected to further investigation to uncover underlying patterns or characteristics.

**Figure 4:**
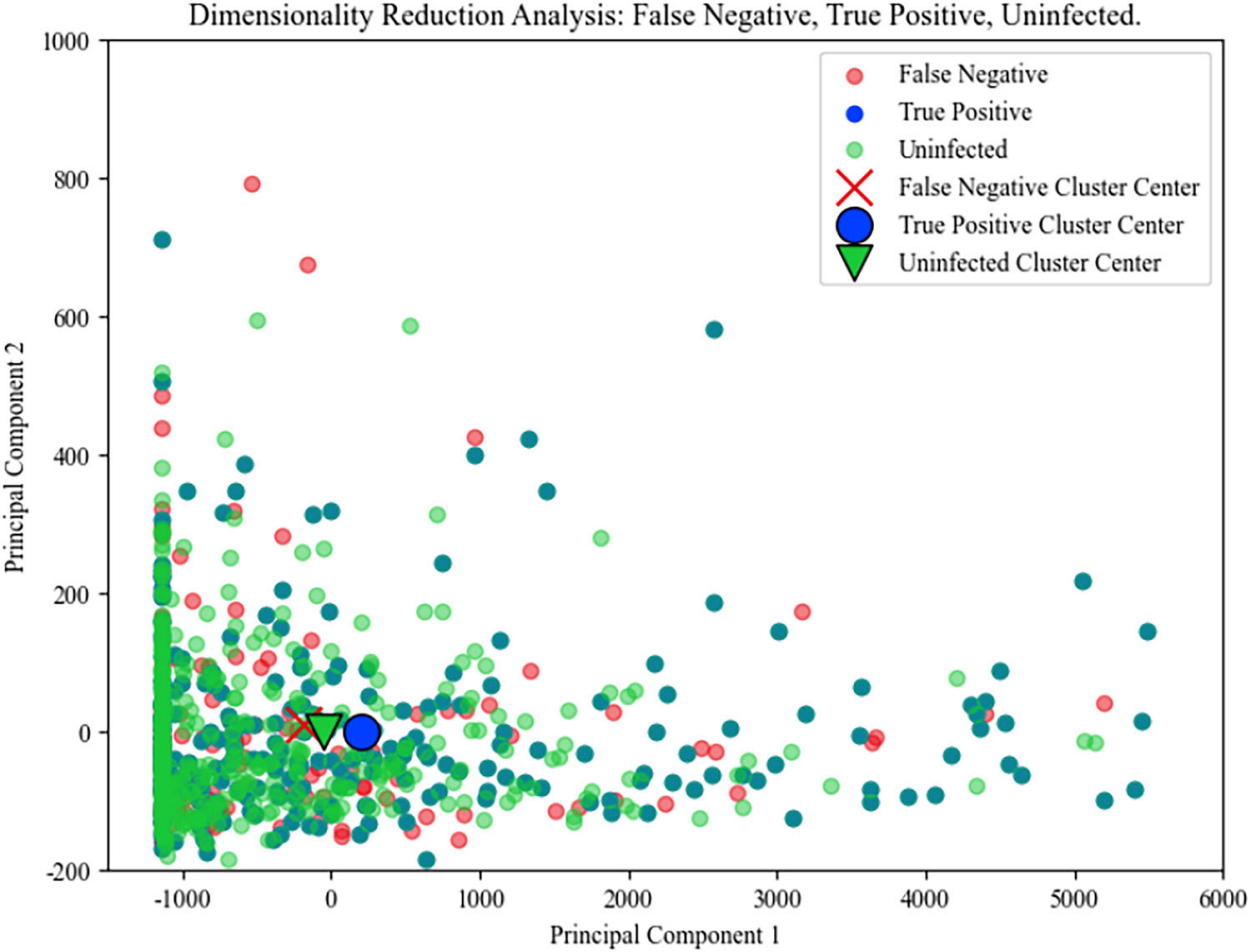
Analysis of the missed data.

Utilizing principal component analysis (PCA), we reduced the feature values of these false‐negative samples to two dimensions. This reduction facilitated a clearer visual representation in a scatter plot, as depicted in Figure 4. In this plot, each point corresponds to a case in the test set. The red points, which represent false‐negatives, significantly overlap with the green points, which denote true positive cases. A crucial observation from this plot is the cluster distance: the distance between the cluster of “false‐negative samples” and the “common fever group” is 56.53, whereas the distance between the “false‐negative samples” and the “true positive samples” is substantially greater at 305.89.

### 3.8 Visual Model Validation

To further validate the model's predictive accuracy, we conducted a simulation analysis using two confirmed cases: one with Flu A infection and another with MP infection. These patients, referred to as Patient A (Flu A case) and Patient B (MP case), were randomly selected for this analysis. The original clinical and laboratory data for these patients are detailed in Table 4.

**Table 4:** Patient information and laboratory indicators.

	Patient A	Patient B
Sex	Female	Male
Age	88	10
White‐cell count (×10^9^/L)	3.8	4.65
Red‐cell count (×10^12^/L)	2.61	4.98
Hemoglobin (g/L)	84	143
Hematocrit (%)	24.8	43.9
Mean corpuscular volume (fL)	94.9	88.2
Mean corpuscular hemoglobin (pg)	32.2	28.7
Mean corpuscular hemoglobin concentration (g/L)	339	326
Platelet (×10^9^/L)	112	276
Standard deviation in red cell distribution width (fL)	56.3	41
Coefficient variation of red blood cell volume distribution width (%)	15.9	12.7
Platelet distribution width (fL)	16.8	16.3
Mean platelet volume (fL)	12.4	9.3
Platelet larger cell ratio (%)	41.3	21.7
Thrombocytocrit (%)	0.139	0.257
Neutrophil count (×10^9^/L)	2.71	2.37
Lymphocyte count (×10^9^/L)	0.87	1.9
Monocyte count (×10^9^/L)	0.22	0.38
Eosinophil count (×10^9^/L)	0	0
Basophil count (×10^9^/L)	0	0
Neutrophil ratio (%)	71.1	50.9
Lymphocyte ratio (%)	23	41
Monocyte ratio (%)	5.8	8.1
Eosinophil ratio (%)	0.1	0
Basophil ratio (%)	0	0
Immature granulocyte count (×10^9^/L)	0.04	0
Immature granulocyte ratio (%)	1	0.1
High fluorescence intensity cell count (%)	0.03	0.09
High fluorescence intensity cell ratio (%)	0.8	2
C‐reactive protein (mg/L)	78.79	0.62

**Figure 5:**
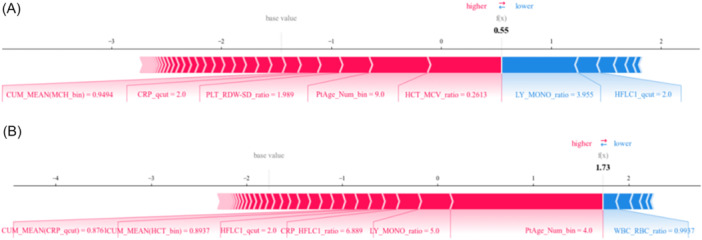
The shap of model prediction score. (A) Patient A. (B) Patent B.

To provide a deeper understanding of the model's decision‐making process for these specific cases, we generated SHapley Additive exPlanations (SHAP) plots, which are presented in Figure 5. SHAP plots offer insightful visual representations of how each feature in the patient's data contributes to the model's predictive outcome. For Patient A, the model calculated a 92.02% probability of a Flu A diagnosis, whereas for Patient B, it predicted a 98.53% probability of an MP infection. These high probabilities demonstrate a strong concordance between the model's predictions and the confirmed clinical diagnoses of these patients.

## 4 Discussion

China's more stringent and prolonged nonpharmaceutical intervention (NPI) measures [14, 15] have brought global attention to the postlockdown influenza scenario in China [16]. In response to this public health challenge, our team focused on developing a predictive model tailored to identify common respiratory virus infections, specifically Flu A and MP, in patients [17, 18].

The CBC test is a fundamental and widely utilized tool in primary clinical screening [19]. Parameters such as WBCs, neutrophils, lymphocytes, and monocytes are crucial, as they can quickly provide insights into a patient's pathological and immune status in response to various diseases [20]. However, in light of the recent surge in MP infections, which predominantly affect children, it becomes imperative to consider the variability in haematological reference ranges between children and adults [21, 22].

Accordingly, in our model, we adopted a categorization approach for each blood cell analysis indicator. These were classified into three distinct categories, namely, “low,” “medium,” and “high,” on the basis of established reference ranges. This classification was guided by the “Children's CBC test Reference Range” and the “Chinese Adult CBC test Reference Range” per the WS/T779‐2021 guidelines. This stratified approach is crucial for ensuring that the model accurately accounts for age‐related variations in blood cell counts, thereby minimizing any potential biases or inaccuracies [23]. This categorization not only enhances the model's diagnostic precision but also significantly contributes to its stability and reliability, especially when dealing with a paediatric population that exhibits distinct haematological profiles compared with adults [24].

In an analogous study, Chang et al. investigated the clinical characteristics of hospitalized children with community‐acquired pneumonia and respiratory infections. Their objective was to predict six common respiratory pathogens [25]. While their model had the advantage of predicting a broader range of pathogens, the methodology presented some limitations. Notably, there was an imbalance in the number of cases for each pathogen during data collection, which could introduce bias into the model learning process. Another critical aspect that differed from our approach was their ability to handle blood cell analysis data. Chang et al. did not implement feature binning for these data; instead, they relied on numerous continuous variables. This approach can potentially diminish the model's robustness, as subtle numerical variations in continuous variables might lead to different prediction outcomes.

In contrast, our study placed significant emphasis on enhancing the stability and robustness of the model [26]. To achieve this goal, we incorporated feature binning in our analysis. This method involves categorizing continuous variables into discrete groups, thereby reducing the impact of minor numerical fluctuations on the model's predictions. Such categorization can lead to more consistent and reliable outcomes, especially in a clinical setting where precision is paramount.

Moreover, to further validate the stability of our model, we conducted PSI tests. The PSI is a metric used to measure the stability of a model by comparing the distribution of predicted probabilities between the training and test sets. These PSI values all fall within the 0–0.1 range, indicating good stability across all three classes, which supports the robustness and generalizability of our model [27], a crucial aspect in ensuring that it performs reliably over time and across various patient populations. This level of stability is particularly important in the dynamic field of medical diagnostics, where models must adapt to new data while maintaining consistent performance.

In addition to basic analysis, our study enhanced the utility of each blood cell indicator by transforming it into more informative features [28]. For example, with respect to the WBC count, after binning, we did not stop there. We further analysed its statistical properties, such as the average, total count, highest and lowest values, and percentiles. Each of these properties was then used as a separate new feature in our model.

A key innovative feature we used is the “dynamic cumulative average” of the WBC. This is calculated by taking each case in turn and computing an ongoing average up to that point. This approach helps in understanding how the WBC count changes over time among different cases [29], providing valuable insights into trends and patterns that could be crucial for diagnosis.

In multiclass samples such as ours, there is a risk of overfitting, especially when the sample sizes vary between groups. Overfitting occurs when a model is too closely fitted to the specific data it is trained on, and as a result, it might not perform well on new, unseen data [30]. Even though our sample distribution was relatively balanced, we still took steps to prevent overfitting [31].

The assignment of different weights to the models—LightGBM, CatBoost, and XGBoost—was determined through an iterative experimental process. During this process, we conducted a series of experiments to evaluate the performance of each model on the validation set, specifically focusing on the F1 scores. The weights were assigned based on the relative performance of each model: LightGBM received the highest weight (0.7) due to its consistently superior performance in handling the imbalanced data and achieving the highest F1 score across multiple iterations. XGBoost was assigned a weight of 0.2, reflecting its robust performance but slightly lower effectiveness compared to LightGBM in this specific data set. CatBoost was assigned a weight of 0.1 as it provided complementary insights, particularly in cases where categorical features played a significant role, though its overall performance was slightly lower than the other two models. The use of multiple models with weighted contributions is a form of ensemble learning, which often leads to more robust and generalizable results compared to single‐model approaches. This strategy helps to reduce bias and variance, potentially leading to improved overall performance.

During the training phase of our LightGBM model, we employed two key strategies. First, we set the feature sampling rate at 0.5. This means that in each step of building the model, only half of the features were used. This approach helps the model in not getting too ‘fixated’ on certain features. Second, we incorporated regularization penalty terms. These penalties discourage the model from becoming too complex by overly relying on specific features, further reducing the risk of overfitting [32].

In our study, we utilized the SHAP method to provide clear explanations of how our model predicts specific pathogens on the basis of individual patient data [33, 34]. This method is crucial for offering both global and local interpretations of the model's functionality. Global explanations provide an overall understanding of how the model works, whereas local explanations provide detailed insights into specific patient predictions. By employing SHAP, we can present the model's workings to clinicians in a manner that is both comprehensive and comprehensible [35].

As a challenge that emergency departments must contend with every winter, how to effectively reduce costs and achieve more efficient manpower allocation has always been an important issue in hospitals. For approximately 1 year, we applied the model in cooperation with the emergency department. Young patients without underlying disease presenting with fever may first undergo routine blood tests at the visit. After the blood test results are obtained, patients can discuss the findings with the clinicians according to the test results and the preliminary judgement of the model. The accuracy of the model is high, and it can effectively divert patients, thereby reducing the pressure on emergency treatment, effectively decreasing the waiting time of patients, and reducing the cost of medical treatment for patients.

With respect to the accessibility of our data and findings, researchers interested in further exploring or validating our work can directly contact the authors to request access to the datasets. We are committed to sharing the results reported in this paper, including individual participant data in a deidentified format. This includes text, tables, figures, and appendices and will be made available for academic and research purposes upon reasonable request.

Despite the strengths of our study, it is important to recognize certain limitations. One key constraint is that it is a single‐centre study. Another limitation arises from the fact that the majority of our data come from patients visiting the emergency department. While this provides a rich source of acute cases, it also presents challenges in long‐term patient tracking. As a result, our model lacks comprehensive data regarding the outcomes of these diseases. This absence of follow‐up data means that we are unable to incorporate disease progression or patient recovery trajectories into the model.

## 5 Conclusion

Our study successfully developed a predictive model for classifying and predicting respiratory pathogenic infections in the post‐COVID‐19 era. This model uses baseline patient data and blood cell analysis indicators to make predictions. The accurate prediction of respiratory infections can aid clinicians in enhancing diagnostic efficiency and accuracy. This improvement can lead to reduced medical expenses by minimizing unnecessary tests and treatments. Additionally, the model's ability to speed up the diagnostic process is particularly beneficial in a clinical setting, where timely decision‐making can have profound impacts on patient outcomes. Ultimately, the integration of this model into clinical workflows has the potential to significantly increase the quality of health care services, offering a more streamlined and precise approach to diagnosing respiratory infections in the postpandemic landscape.

## Author Contributions

**Tian‐ning Li:** conceptualization, data curation, formal analysis, methodology, project administration, validation, writing – original draft, writing – review and editing. **Yan‐hong Liu:** conceptualization, funding acquisition, investigation, supervision, validation. **Kwok‐Leung Yiu:** resources, software, supervision, validation, visualization. **Lu Liu:** investigation, methodology, project administration, software. **Meng Han:** formal analysis, project administration, validation, visualization. **Wei‐jia Ma:** resources, supervision, validation, visualization, writing – review and editing. **Chun‐lei Zhou:** conceptualization, data curation, formal analysis, funding acquisition, software, visualization, writing – original draft, writing – review and editing. **Hong Mu:** conceptualization, data curation, funding acquisition, investigation, visualization, writing – review and editing.

## Data Availability

The authors have nothing to report.
